# Electrophoretically Co-Deposited Collagen–Lactoferrin Membranes with Enhanced Pro-Regenerative Properties for Oral Soft Tissue Regeneration

**DOI:** 10.3390/ijms242417330

**Published:** 2023-12-10

**Authors:** Artem Antoshin, Mikhail Gostev, Yana Khristidis, Aliia Giliazova, Sergei Voloshin, Nataliia Blagushina, Olga Smirnova, Ekaterina Diachkova, Elena Istranova, Anna Usanova, Nikolai Solodov, Alexey Fayzullin, Elena Ivanova, Elena Sadchikova, Milena Noelia Vergara Bashkatova, Olga Drakina, Svetlana Tarasenko, Peter Timashev

**Affiliations:** 1 Institute for Regenerative Medicine, Sechenov University, 8-2 Trubetskaya St., 119048 Moscow, Russia; antoshin_a_a@staff.sechenov.ru (A.A.); khristidis_ya_i@staff.sechenov.ru (Y.K.); gilyazova_a_n@staff.sechenov.ru (A.G.); voloshin_s_yu@staff.sechenov.ru (S.V.); smirnova_o_a_2@staff.sechenov.ru (O.S.); istranova_e_v@staff.sechenov.ru (E.I.); usanova_a_p@staff.sechenov.ru (A.U.); fayzullin_a_l@staff.sechenov.ru (A.F.); ivanova_e_i_1@staff.sechenov.ru (E.I.); 2 Department of Oral Surgery, Borovskiy Institute of Dentistry, Sechenov University, 8-2 Trubetskaya St., 119048 Moscow, Russia; gostev_m_s@staff.sechenov.ru (M.G.); blagushina_n_a@staff.sechenov.ru (N.B.); dyachkova_e_yu_1@staff.sechenov.ru (E.D.); solodov-niko@yandex.ru (N.S.); tarasenko_s_v@staff.sechenov.ru (S.T.); 3 Institute of Gene Biology, Russian Academy of Sciences, 34/5 Vavilov St., 119344 Moscow, Russia; e.r.sadchikova@gmail.com; 4 Department of Operative Surgery and Topographic Anatomy, Sechenov University, 8-2 Trubetskaya St., 119048 Moscow, Russia; milenacatcat@yandex.ru (M.N.V.B.); drakina_o_v@staff.sechenov.ru (O.D.); 5 World-Class Research Center “Digital Biodesign and Personalized Healthcare”, Sechenov University, 8-2 Trubetskaya St., 119048 Moscow, Russia

**Keywords:** collagen, lactoferrin, electrophoretic deposition, SBA-EPD, vestibuloplasty, free gingival graft harvesting, membrane

## Abstract

The quality of soft tissue defect regeneration after dental surgeries largely determines their final success. Collagen membranes have been proposed for the healing of such defects, but in some cases, they do not guarantee a sufficient volume of the regenerated tissue and vascularization. For this purpose, lactoferrin, a protein with natural pro-regenerative, anti-inflammatory, and pro-angiogenic activity, can be added to collagen. In this article, we used a semipermeable barrier-assisted electrophoretic deposition (SBA-EPD) method for the production of collagen–lactoferrin membranes. The membrane structure was studied by SEM, and its mechanical properties were shown. The lactoferrin release kinetics were shown by ELISA within 75 h. When tested in vitro, we demonstrated that the collagen–lactoferrin membranes significantly increased the proliferation of keratinocytes (HaCaT) and fibroblasts (977hTERT) compared to blank collagen membranes. In vivo, on the vestibuloplasty and free gingival graft harvesting models, we showed that collagen–lactoferrin membranes decreased the wound inflammation and increased the healing rates and regeneration quality. In some parameters, collagen–lactoferrin membranes outperformed not only blank collagen membranes, but also the commercial membrane Mucograft^®^. Thus, we proved that collagen–lactoferrin membranes produced by the SBA-EPD method may be a valuable alternative to commercially used membranes for soft tissue regeneration in the oral cavity.

## 1. Introduction

The quality of the oral tissue regeneration after dental surgeries, for a number of them, plays a crucial role affecting their final success. Regarding oral tissue regeneration, guided bone regeneration is the most commonly mentioned in the literature [1], but another important aspect is the regeneration of soft tissue, particularly the mucosa. Mucosal regeneration, for example, may be required for surgeries such as vestibuloplasty and free gingival graft (FGG) harvesting in the hard palate region [2,3]. Vestibuloplasty is a surgical procedure that aims to increase the depth of or reshape the space between the mucosa of the inner surface of the cheek or lip and the alveolar bone. A free gingival graft is usually harvested from the hard palate in order to increase the volume of gingival tissue in another (deficient) area of the mouth.

Particularly, FGG from the hard palate may be harvested for the purpose of vestibuloplasty (Clark’s modification) to cover an exposed alveolar periosteum [4]. However, for covering large defects, the area of the FGG available for harvesting may be insufficient [5,6]. In this case, if the periosteum heals naturally, the process will be long and painful for the patient; it may also be accompanied by infectious complications, scarring, and deformation of the newly formed mucosa [7]. Similarly, when FGG is harvested, the connective tissue layer covering the hard palate periosteum is exposed, and in the case of natural healing, it is extremely painful for the patient in the postoperative period and can be associated with infectious complications [8,9].

Both of these cases require the use of auxiliary methods to regenerate the deficient mucosal cover. One of the possible approaches is the use of membranes (also called matrices, scaffolds). For this purpose, the membranes of a biological (allogenic or more often xenogenic) nature and based on collagen (Col) are the most favored. To date, a large number of collagen-containing membranes are available on the market. The increased interest in collagen membranes is due to their properties: biocompatibility, biodegradation, strength, elasticity, and pro-regenerative properties [10,11,12].

In particular, the collagen-based membrane Mucograft^®^ has been widely used as a “gold standard” for enlarging the area of the attached gingiva. However, according to the data of recent studies, its use in extensive defects of the oral mucosa does not always lead to the necessary soft tissue volume regeneration, while histological analysis has shown insufficient vascularization of the newly formed tissues [13].

One of the possible solutions to improve the healing properties of collagen membranes may be the addition of biologically active substances (BAS) into their structure [14]. Among such BAS, a promising candidate for the needs of dentistry is lactoferrin (LF), a natural protein with powerful antimicrobial, pro-regenerative, and angiogenic activities [15]. Indeed, a number of preclinical and clinical trials have proved that LF may be valuable for epithelial barrier and connective tissue regeneration [16], which is especially relevant for oral soft tissue regeneration.

We have previously demonstrated the potential of semipermeable barrier-assisted electrophoretic deposition (SBA-EPD) for the preparation of collagen membranes for dental applications [17]. SBA-EPD allows for producing collagen membranes with controlled and reproducible mechanical and biodegradation properties that can be varied in a wide range. In addition, the EPD principle allows for the simultaneous incorporation of other substances into a formed collagen membrane (including BASs) [18].

Therefore, in this work, we applied the collagen–lactoferrin electrophoretic co-deposition principle for the production of membranes for oral soft tissue regeneration. The beneficial effects of LF incorporation into collagen membranes have been shown in vitro on two cell types (keratinocytes and fibroblasts) and in vivo in animal experiments (vestibuloplasty and free gingival graft harvesting models). According to our knowledge and literature analysis, this is the first work to apply the EPD principle for the production (co-deposition) of collagen–lactoferrin membranes. Furthermore, collagen has already been combined with lactoferrin to make membranes for guided bone regeneration [19,20] (in vitro studies) or skin healing [21] (in vitro and in vivo studies). However, a collagen–lactoferrin (Col-LF) composition has not yet been tested for the regeneration of soft tissues in the oral cavity.

## 2. Results

### 2.1. Structural and Physicochemical Properties

**Figure 1:**
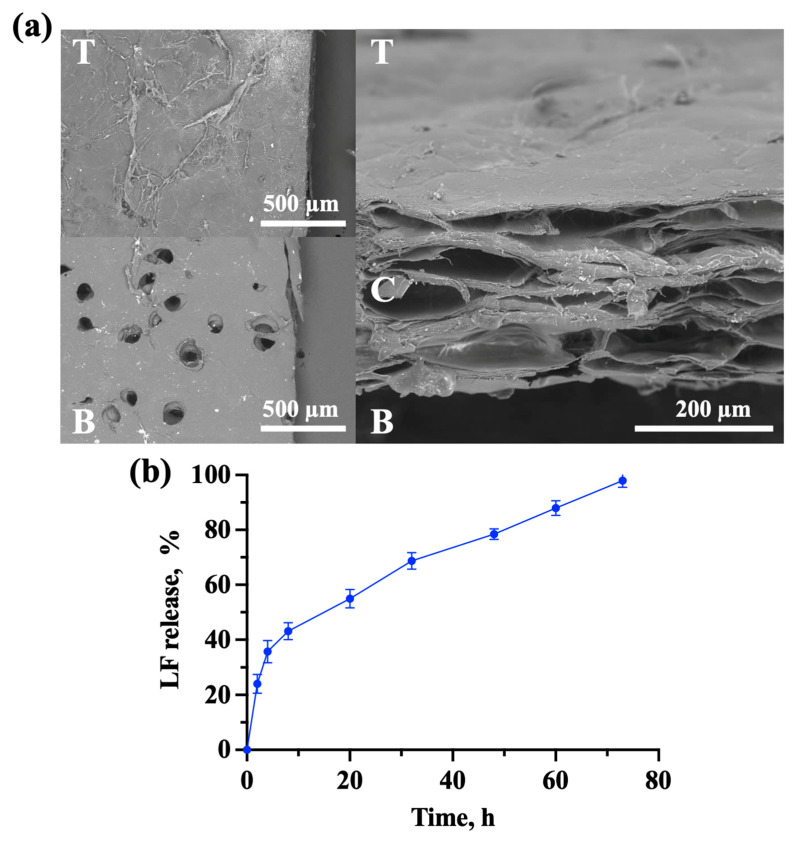
Structural and chemical properties of Col-LF membranes. (**a**) Top (T), bottom (B), and cross (C) structure of membranes investigated by SEM; (**b**) kinetics of LF release from Col-LF membranes over time, as a percentage of total LF content in the membrane.

The structure of the Col-LF membranes had an interface design (Figure 1a). Scanning electron microscopy (SEM) photographs show that their top side was solid and unperforated, while the bottom side was perforated; the central part of the membranes (section) was layered and porous. The pores of the central part were not open: they were located between the deposited collagen layers and had no exit to the surface, except for the layers located close to the bottom side of the membrane; in this case, the “openness” of the pores was provided by artificially created mechanical perforations.

The kinetics of LF release from the membranes (Figure 1b) were relatively stable over the 3 days: it was shown that about 40% of the LF is released in the first 10 h after membrane placement in the liquid medium and, thereafter, this rate slowed down but remained stable, and the LF was released almost completely (98 ± 2%) by the 75th hour of the observation.

When analyzing and comparing the physical properties of the Col and Col-LF membranes, a statistically significant difference was found only in their thickness, while no significant differences were found in the swelling, shrinkage temperature, or mechanical properties (Young’s modulus and strain at fracture) (Table 1).

### 2.2. In Vitro Testing of Membrane Biocompatibility

**Figure 2:**
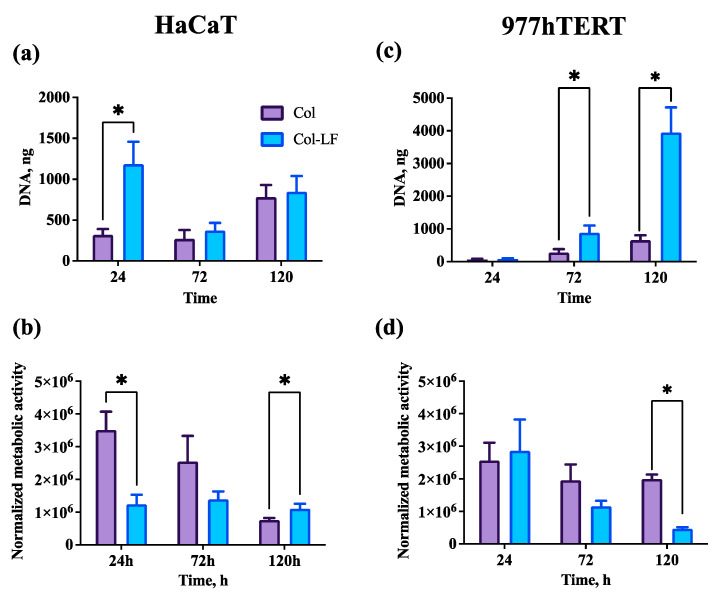
Contact biocompatibility of collagen and collagen–lactoferrin membranes. (**a**) Proliferative activity of HaCaTs; (**b**) normalized metabolic activity of HaCaTs; (**c**) proliferative activity of 977hTERTs; (**d**) normalized metabolic activity of 977hTERTs. Results are presented as mean ± SD. * *p* < 0.05.

The contact (direct) effect of the produced Col-LF membranes in comparison to the Col membranes was shown on the HaCaT (keratinocytes) and 977hTERT (fibroblasts) cell types (Figure 2). It was shown that the HaCaT cell line had a statistically higher amount of DNA on membranes with lactoferrin relative to blank collagen membranes by 24 h of culturing (Figure 2a) [1184 ± 274 ng vs. 320 ± 72 ng], which was simultaneously associated with a decrease in normalized metabolic activity (Figure 2b) [(1.2 ± 0.3) × 10^6^ vs. (3.5 ± 0.6) × 10^6^]. At 72 h, the amount of DNA for the Col-LF group decreased and equaled the Col group, while, by 120 h, it increased slightly for both groups. The normalized metabolic activity for the Col-LF group gradually increased, and by 120 h it was statistically significantly higher than that for the Col group.

The proliferative pattern was different in the 977hTERT cell line relative to the HaCaT cell line (Figure 2c). The proliferative activity growth rate was slower in the two groups, but by 120 h, the difference in the amount of DNA was quite large between the Col-LF and Col membranes (3948 ± 772 ng vs. 656 ± 150 ng, respectively). The change in the normalized metabolic activity was inversely proportional to the rate of cell growth in the groups (Figure 2d), and for the LF-Col group, starting from 72 h, it was the lesser the greater the rate of cell proliferation was.

### 2.3. Animal Experiments

#### 2.3.1. Postoperative Evaluation

**Figure 3:**
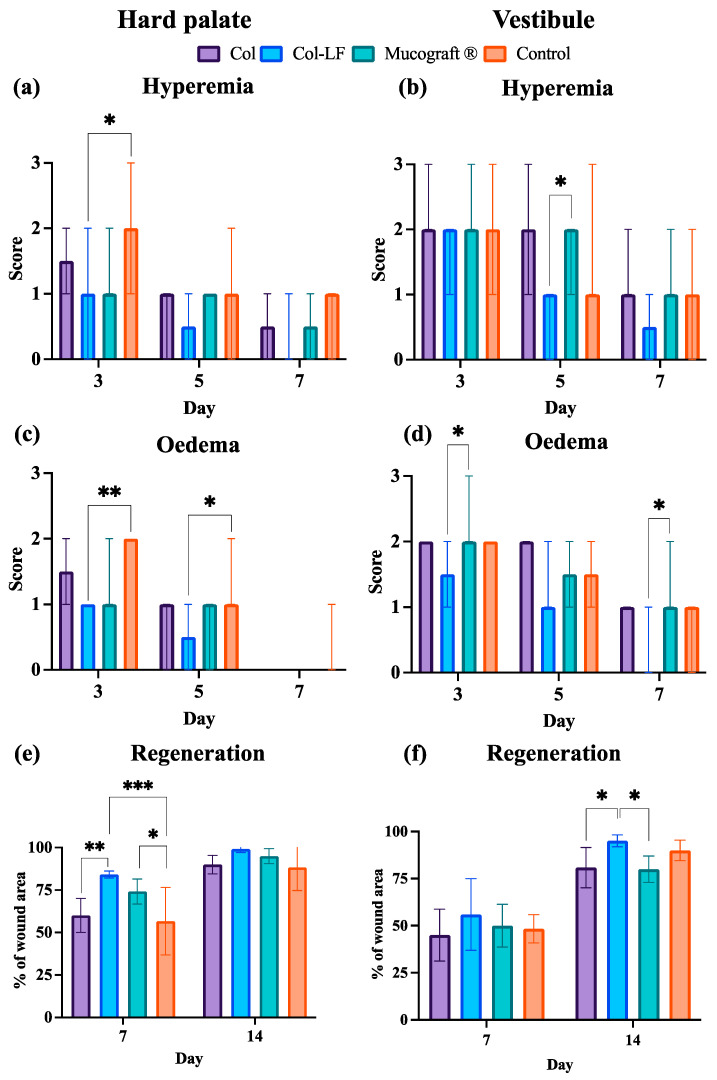
Postoperative evaluation of oral soft tissues. Results are presented as median and min/max (**a**–**d**), and as mean ± SD (**e**,**f**). * *p* < 0.05; ** *p* < 0.01; *** *p* < 0.001.

The results of the oral soft tissue assessment of the operated-on animals in different groups are shown in Figure 3.

The level of hyperemia on day 3 on the hard palate (Figure 3a) was statistically significantly less in the Col-LF membrane group compared to the control group, while on the following days this score equalized for all other groups and gradually decreased. In the vestibuloplasty area (Figure 3b), the hyperemia was statistically significantly less on day 5 in the Col-LF group compared to the Mucograft^®^ group, but by day 7, the statistically significant difference between the groups disappeared. Nevertheless, the Col-LF group tended to have less hyperemia overall compared to the others.

The most pronounced oedema in the area of the hard palate was observed in the control group on all days, where healing occurred naturally (Figure 3c). Interestingly, on days 3 and 5, only the Col-LF group had significantly less oedema than the control group. A similar trend in the reduction in oedema for the Col-LF group was noted in vestibuloplasty (Figure 3d), and oedema was significantly lower in the Col-LF group compared to the Mucograft^®^ application group on both days 3 and 7.

The tissue regeneration of the defect area on the hard palate and in the vestibulum was assessed by measuring the area of newly formed tissue at 7 and 14 days postoperatively as the severity of oedema and hyperemia progressively decreased. In the FGG hard palate harvesting group at day 7 (Figure 3e), better regeneration was noted in the Col-LF membrane group (84 ± 2%) compared to the Col and control groups (60 ± 10% and 57 ± 20%, respectively), while the Mucograft^®^ membrane (74 ± 7%) was only more effective than the control group. By day 14, the statistically significant differences between all groups disappeared.

In contrast, in the vestibuloplasty group (Figure 3f), a statistically significant difference between the groups appeared only by day 14 postoperatively, and the highest degree of regeneration was again shown for the Col-LF group (95 ± 3%), which was statistically significantly greater than that seen in the Col and Mucograft^®^ groups (81 ± 11% and 80 ± 7%).

#### 2.3.2. Histological Analysis (Hard Palate)

##### Collagen Group

**Figure 4:**
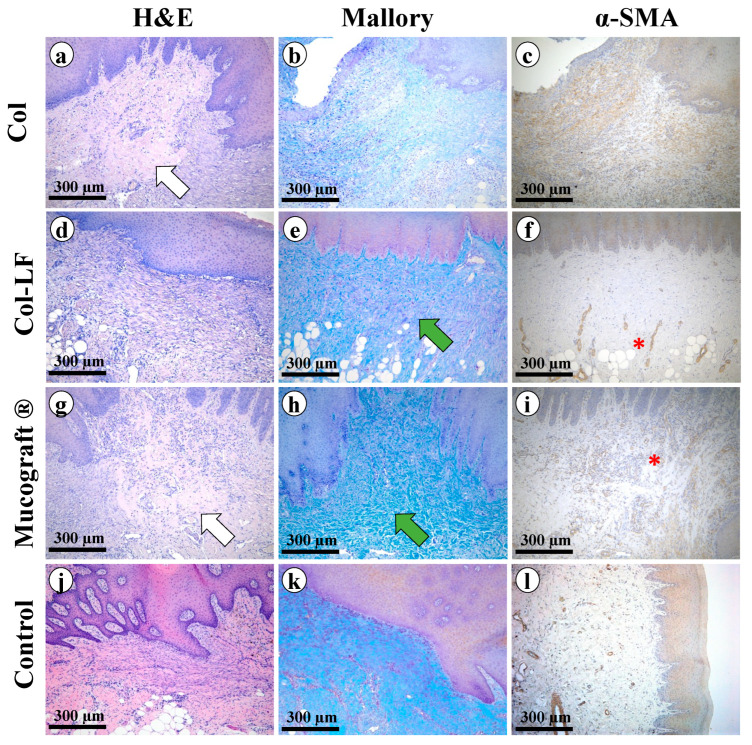
Morphological analysis of hard palate regeneration sites after free gingival graft harvesting. Groups: Col (**a**–**c**), Col-LF (**d**–**f**), Mucograft (**g**–**i**), control (**j**–**l**). Magnification ×100. H&E, Mallory, α-SMA staining. Remaining fragments of the membrane (white arrow), thick collagen fibers of mature granulation tissue (green arrows), blood vessel loops (*) indicating neoangiogenesis.

The regenerated defect was lined with stratified squamous non-keratinized epithelium that, in some zones, was necrotic. Signs of exudation and microcirculatory disorders were determined near the necrosis zones (Figure 4a). In the submucosa and the underlying tissues, the granulation tissue, in large quantities, as well as newly formed connective tissue built of multidirectional bundles of thin collagen fibers were determined. Between the collagen fibers, there were fibroblasts and immune cells (segmented neutrophils, macrophages, plasmocytes). Also, in this area, there was a moderate number of newly formed vessels with thin, not fully formed walls and a lining of thinned endothelium. In some places, the vessels were surrounded by moderately pronounced infiltration represented by lymphocytes, macrophages, and single eosinophils.

At Mallory staining of the submucosa (Figure 4b), multidirectional bundles of thin collagen fibers stained in light blue color were determined; the nuclei of fibroblasts were reddish in color. The immunohistochemical reaction to α-smooth muscle actin (α-SMA) with antibodies detected moderate expression in fibroblasts (++) as well as in the muscularis vasculature (++) (Figure 4c). Myofibroblasts formed a thick layer of α-SMA-positive cells under the epithelium at the implantation site.

##### Collagen–Lactoferrin Group

The regenerated defect was lined with stratified squamous non-keratinized epithelium without signs of dystrophy. Necrotic changes in the epithelium were not detected (Figure 4d). In the submucosa and underlying tissues, there was a small amount of granulation tissue, and newly formed connective tissue was determined. Also, in this area, there were fibroblasts with elongated ovoid basophilic nuclei, as well as immune cells in small numbers. It is worth noting the abundant vascularization in the implantation area: a large number of newly formed vessels with a multilayer-formed thick wall and endothelium lining of the usual structure were determined. In some places, vessels were surrounded by a weak infiltration of lymphocytes, macrophages, and single eosinophils.

At Mallory staining of the submucosa (Figure 4e), unidirectional bundles of collagen fibers of a dark blue color were determined; the fibroblast nuclei were reddish in color, and the bundles of muscle fibers were stained yellowish. The immunohistochemical reaction to α-SMA with antibodies detected weak expression in fibroblasts (+) and moderate expression in vascular muscle (++) (Figure 4f).

##### Mucograft^®^ Group

The regenerated defect was lined with significantly thickened stratified squamous non-keratinized epithelium. No areas of epithelium necrosis were found (Figure 4g). In the middle part of the defect, there was a collagen matrix consisting of unidirectional fibers that were significantly thickened due to hyalinosis. Between the fibers, there were thin layers of granulation tissue, immune cells (segmented neutrophils, macrophages, plasmocytes), and a small number of fibroblasts. More granulation tissue was present at the periphery of the implant, with newly formed vessels with thin walls and lined with formed endotheliocytes. The signs of exudation and microcirculatory disorders were weak.

At Mallory staining of the implantation area (Figure 4h), there were elements of collagen material consisting of unidirectional collagen fibers that were significantly thickened and stained bright blue; the fibroblast nuclei were reddish in color, and the immune cell nuclei were reddish-purple in color. The immunohistochemical reaction to α-SMA with antibodies revealed moderate expression in myofibroblasts as well as in myocytes of the vascular wall (++) (Figure 4i).

##### Control Group

The regenerated defect was lined with significantly thickened or thinned stratified squamous non-keratinized epithelium (Figure 4j). In the submucosa, collagen fibers were practically unidirectional, and a small number of fibroblasts and thin-walled vessels were detected. As well, areas of necrosis were detected that were densely infiltrated and surrounded by a wall of immune cells (predominantly segmented neutrophils with a small number of macrophages and plasmocytes). There were also signs of exudation and microcirculatory disorders near the necrosis zones.

Mallory staining revealed unidirectional parallel bundles of collagen fibers stained blue, while the fibroblast nuclei were reddish in color and immune cell nuclei were reddish-purple in color (Figure 4k). The immunohistochemical reaction to α-SMA with antibodies showed weak expression in myocytes of the vessel walls (+) (Figure 4l).

#### 2.3.3. Histological Analysis (Vestibule)

##### Collagen Group

**Figure 5:**
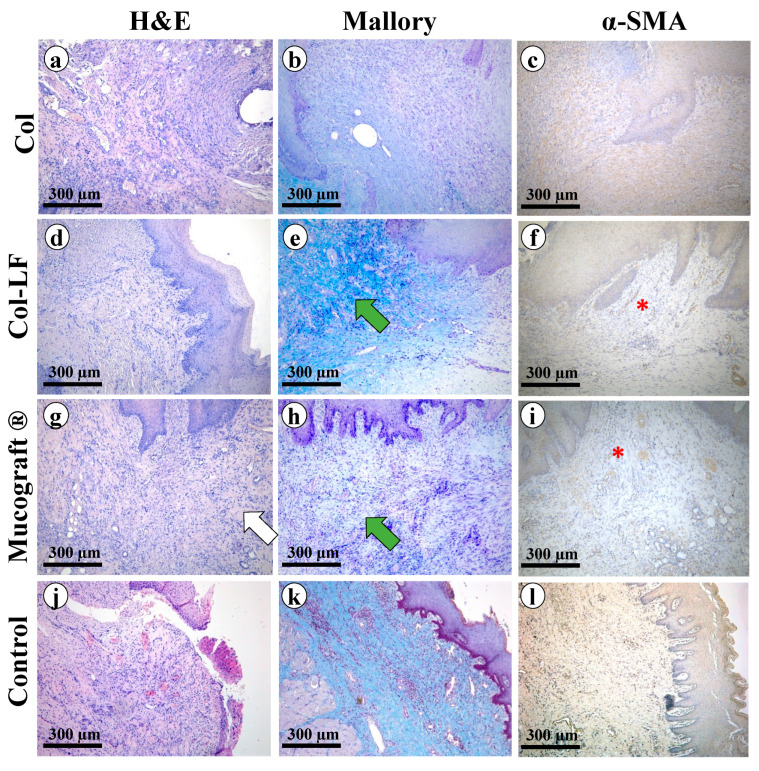
Morphological analysis of the regeneration sites after vestibuloplasty. Groups: Col (**a**–**c**), Col-LF (**d**–**f**), Mucograft (**g**–**i**), control (**j**–**l**). Magnification ×100. H&E, Mallory, α-SMA staining. Remaining fragments of the membrane (white arrow), thick collagen fibers of mature granulation tissue (green arrows), blood vessel loops (*) indicating neoangiogenesis.

The regenerated defect was lined with stratified squamous non-keratinized epithelium that, in some areas, was necrotic. Signs of exudation and microcirculatory disorders were detected near the necrosis zones (Figure 5a). In the submucosa and the underlying tissues, granulation tissue was determined in large quantities, as well as newly formed connective tissue built of thin collagen fibers. Between collagen fibers, there were fibroblasts and immune cells in large numbers (segmented neutrophils, macrophages, plasmocytes). Newly formed vessels with thin, not fully formed walls and thinning endothelium lining were also detected in this area. In some places, the vessels were surrounded by moderately expressed infiltration represented by lymphocytes, macrophages, and single eosinophils.

At Mallory staining, the multidirectional bundles of thin collagen fibers were stained in a light blue color, and the nuclei of fibroblasts were reddish in color (Figure 5b). The immunohistochemical reaction to α-SMA with antibodies detected moderate expression in fibroblasts (++) as well as in the muscularis vasculature (++) (Figure 5c). Myofibroblasts formed a thick layer of α-SMA-positive cells under the epithelium at the implantation site.

##### Collagen–lactoferrin Group

The regenerated defect was lined with stratified squamous non-keratinized epithelium and no necrotic changes were detected (Figure 5d). In the submucosa, there were fibroblasts with elongated ovoid basophilic nuclei, a small number of immune cells, and granulation tissue. A large number of newly formed vessels with a fully formed thick wall of several layers and endothelial lining of the usual structure were revealed.

At Mallory staining (Figure 5e), unidirectional bundles of collagen fibers of a dark blue color were determined; the fibroblast nuclei were reddish in color, and the muscle fiber bundles were yellowish in color. The immunohistochemical reaction to α-SMA with antibodies detected weak expression in fibroblasts (+) and moderate expression in vascular muscularis (++) (Figure 5f).

##### Mucograft^®^ Group

The regenerated defect was lined with stratified squamous non-keratinized epithelium and no necrotic changes were detected (Figure 5g). The middle part of the implantation area contained collagen matrix consisting of unidirectional collagen fibers that were significantly thickened due to hyalinosis. Between the fibers, there were thin layers of granulation tissue, immune cells (segmented neutrophils, macrophages, plasmocytes), and fibroblasts in a small amount. More granulation tissue was present at the periphery of the implant with newly formed vessels with thin walls and lined with formed endotheliocytes. The signs of exudation and microcirculatory disorders were weak.

At Mallory staining (Figure 5h), the collagen material elements consisting of unidirectional collagen fibers were stained bright blue. In some areas, the forming thin collagen fibers had a light blue coloration, the fibroblast nuclei were reddish in color, and the nuclei of immune cells were reddish-purple in color. The immunohistochemical reaction to α-SMA with antibodies showed moderate expression in myo- and fibroblasts, as well as in myocytes of the vascular wall (++) (Figure 5i).

##### Control Group

The regenerated defect was lined with stratified squamous non-keratinized epithelium that in some areas was thin or thickened compared to normal epithelium and formed outgrowths penetrating the submucosa (Figure 5j). In some areas, the epithelium was necrotized. In the submucosa, there were also foci of necrosis infiltrated with neutrophils and surrounded by shafts of segmented neutrophils, lymphocytes, and plasmocytes. Signs of exudation and microcirculatory disorders were determined near the necrosis zones. There were also zones of granulation tissue growth, and in some areas, connective tissue was formed. Collagen fibers in most areas had the same direction, and a small number of fibroblasts was determined. Thin-walled vessels were present, and a moderate infiltration of neutrophils and lymphocytes was determined around them.

Mallory staining revealed unidirectional, parallel bundles of bluish-colored collagen fibers in the submucosa (Figure 5k). The nuclei of fibroblasts as well as inflammatory cells were dark red in color, and the muscle fibers were yellowish in color. The immunohistochemical reaction to α-SMA with antibodies revealed weak expression in the muscular wall of vasculature (+) (Figure 5l).

#### 2.3.4. Morphometric Analysis

**Figure 6:**
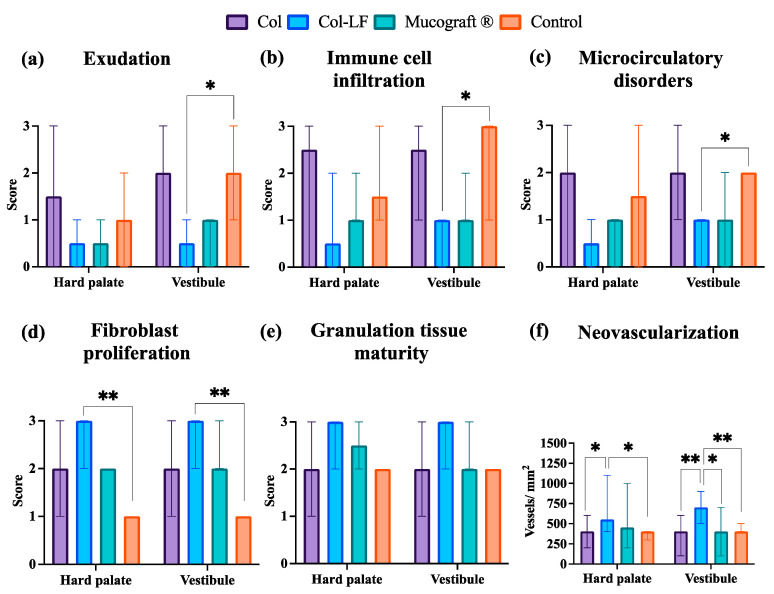
Statistical analysis of morphologic signs of inflammation (**a**–**c**) and regeneration (**d**–**f**) at the implantation sites. Values are presented as median and min/max. * *p* < 0.05, ** *p* < 0.01.

After conducting semiquantitative and quantitative analyses of morphologic changes in defects of vestibuloplasty and FGG harvesting models, the following differences were found (Figure 6). After vestibuloplasty, there was a statistically significant difference between the Col-LF group and the control group in terms of inflammatory reactions (Figure 6a–c): exudation (median 0.5 [0; 1] vs. 2 [1; 3]), immune cell infiltration (median 1 [0; 1] vs. 3 [1; 3]), and microcirculatory changes (median 1 [0; 1] vs. 2 [2; 2]). No other group except Col-LF could decrease the inflammation in comparison to control.

Regarding regeneration (Figure 6d–f), there was the opposite tendency. After both types of operation (vestibuloplasty and FGG harvesting), the fibroblast proliferation was statistically significantly higher in the Col-LF group than in the control group (median 3 [2; 3] vs. 1 [1; 1], respectively). No other group except Col-LF could increase the fibroblast proliferation in comparison to the control group. The more pronounced differences of the Col-LF group from the others were in regards to neovascularization.

In the case of hard palate FGG harvesting, the density of the vessels in the Col-LF group (median 550 [400; 1100] vessels/mm^2^) was statistically significantly different from that of the control group (median value 400 [300; 400] vessels/mm^2^) and of the Col group (median value 400 [200; 600] vessels/mm^2^). In vestibuloplasty surgery, the Col-LF group (median 700 [500; 900] vessels/mm^2^) differed not only from the Col group (median 400 [100; 600] vessels/mm^2^) and the control group (median 400 [400; 500] vessels/mm^2^), but also from the Mucograft^®^ group (median 400 [100; 700] vessels/mm^2^).

## 3. Discussion

Various BASs are added to collagen membranes to improve their functional properties in dental surgery. For guided bone regeneration, bone morphogenetic protein-2, fibroblast growth factor-2, platelet-derived growth factor, and others [14] have been introduced into collagen membranes. For oral soft tissue regeneration [22], platelet-rich fibrin [23] and platelet-derived growth factor [24] have been proposed as additives to collagen membranes. However, most of these studies are preclinical, since the introduction of recombinant growth factors or BASs isolated from blood can be associated with significant regulatory constraints, biosafety wariness, expensiveness, or limitations in the scalability of their commercial production [14,25].

Lactoferrin may be a very valuable alternative BAS to be introduced in collagen membranes for oral soft tissue regeneration. LF is a safe therapeutic agent that has already proved its effectiveness in clinical trials for wound regeneration in patients with skin and ligament injuries [26,27], is available for commercial production in large scales while keeping the final price inexpensive [28,29], and is already used in dentistry as an active ingredient for mouthwashes [30]. In our study, the incorporation of LF into the structure of collagen membranes by SBA-EPD was possible, since collagen and lactoferrin behave similarly in an electric field when dissolved in an acidic medium. Indeed, LF and collagen zeta potentials are both positive in acidic medium [31,32]; therefore, these proteins, together, can co-deposit into a single structure by being attracted to the negatively charged cathode [33].

The LF presence in the membrane after co-deposition was proved by ELISA analysis with specific antibodies. The release kinetics of LF were observed within 75 h: about 40% was released during the first 10 h but, thereafter, the release of the remaining 60% was more stable. This was probably due to a dense packing of collagen membranes [17] together with LF that was evenly distributed over their volume. Indeed, such LF release kinetics from the Col-LF co-deposited membranes formed by the developed SBA-EPD principle differed from methods where collagen membranes were impregnated with LF simply by immersion: LF, which was adsorbed only on the membrane surface, was released within the first 5 h [21].

We chose an interface membrane design [34], where the properties of its top and bottom sides differed from each other: one side was solid and the other side was mechanically perforated. We chose this design based on experience from the development of scaffolds for urethroplasty [35], since the conditions of scaffold functioning are relatively similar. The top (solid) side of the membrane was faced toward the oral lumen with liquid medium (saliva) providing a barrier function and preventing the penetration of food particles and microorganisms into the membrane body. The bottom (perforated) side of the membranes was faced toward the connective tissue in the wound defect: the perforations allowed for the faster penetration of connective tissue and vessels into the membrane body thickness.

Expectedly, the addition of LF to collagen membranes slightly increased their thickness. However, at the level of other physical properties (shrinkage temperature, swelling, and mechanical parameters), the contribution of LF was statistically insignificant. This is so because fibrillar collagen predominantly determines the physical properties of membranes [36], while the contribution of LF in the molecular form at such a ratio with collagen (10:1 for Col and LF by dry mass, respectively) is negligible.

In our in vitro experiments, we used two cell types, keratinocytes and fibroblasts, that are the main components of the oral mucosa [37]. Of note, we used established cell lines (HaCaTs and 977hTERT) in order to minimize the variability in the results (which is typical for primary cultures) and to make our tests maximally standardizable and reproducible [38]. The need for such result reproducibility is due to the fact that LF was reported to influence keratinocytes and fibroblasts ambiguously in the literature: in several studies, LF significantly stimulated the proliferative activity of keratinocytes [39], while in other studies (on healthy cells and a cancer cell line), it could inhibit their growth [40]; in some studies, LF increased fibroblast proliferation [41], while in others, LF inhibited this activity [42]. In our study, LF significantly increased the proliferative activity of both cell types, while the observed decrease in their metabolic activity at high proliferation rates was attributed to contact inhibition due to the restricted area for subsequent cell growth [43].

The anti-inflammatory effects of LF are well known [16], which we also proved by macroscopic postoperative evaluation as well as histologic analysis of the oral mucosa in animals with applied Col-LF membranes compared to other groups. Similar to in vitro tests, there is no consensus in the literature on the effect of LF on the proliferative activity of mucosal cells in vivo. For example, lactoferrin has demonstrated the ability to both increase the proliferative activity of mucosal cells [44,45] and inhibit it [46,47], depending on the dosage; alternatively, when applied to the injured sinus mucosa, it had no pro-regenerative effect on the wound [48].

In our in vivo study, it was shown that LF contained in the membranes increased not only the proliferative activity of fibroblasts in both animal models, but also the vascularization of regenerated tissues compared to the other groups (having the highest value of vessels/mm^2^). Due to better neoangiogenesis, the epithelium necrotic processes were avoided in the Col-LF group compared to the control and Col groups: in them, we assume, the rate of neoangiogenesis was insufficient to meet the need of the growing epithelium [49]. For Mucograft**^®^**, the neovascularization was sufficient, and no epithelial necrosis was observed, but its level was still lower than in the Col-LF group (vestibuloplasty), which may influence the quality of mucosa regeneration in longer timeframes [50].

### Limitations

Despite the observed good regeneration of soft tissues of the oral cavity when using Col-LF membranes, the issue of evaluating the quality of regeneration in the distant postoperative period (5 or more years) is still disputable. In this period, the “volume loss” syndrome of regenerated soft tissues may be observed [2]. Such a long-term experimental design is difficult to realize on a preclinical model, but this fact should be taken into account when planning further clinical trials of this membrane. Other limitations of our study were the small number of animals per group, as well as the relatively non-critical size of the mucosal defect, which could heal naturally.

## 4. Materials and Methods

### 4.1. Collagen–Lactoferrin Membrane SBA-EPD

The schemes of collagen extraction and membrane production were similar to that described in [17]. Briefly, collagen was extracted from frozen bovine Achilles tendons. The tendons were cut into 1 cm-thick pieces, treated with 0.5 M NaCl, homogenized in 0.5 M acetic acid, and supplemented with 0.1% pepsin (Sigma Aldrich, St. Louis, MO, USA) for overnight partial hydrolysis. Then, pepsin was pH-inactivated, and collagen was precipitated with a 12% NaCl solution. The precipitate was redissolved in 0.5 M acetic acid and dialyzed against 0.5 M acetic acid for 3 days. The final collagen concentration in the suspension was determined by gravimetric analysis. After determination of collagen concentration, it was diluted to the required concentration by 0.5 M acetic acid solution.

The LF solution (provided by the Institute of Gene Biology, Russian Academy of Sciences, Moscow, Russia [51]) was prepared in 0.2 M acetic acid and added to the obtained collagen suspension so that the final concentration of collagen was 5 mg/mL and that of LF was 0.5 mg/mL. For the preparation of control (blank, lactoferrin-free) membranes, a collagen solution with a concentration of 5 mg/mL was prepared.

**Figure 7:**
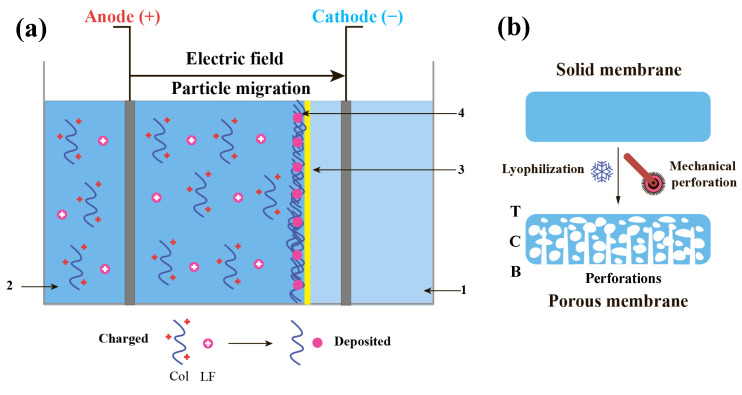
Scheme of collagen–lactoferrin membrane SBA-EPD (**a**) and subsequent post-treatment procedure (**b**). 1—distilled water, 2—collagen–lactoferrin suspension, 3—semi-permeable membrane, 4—deposited membrane. T—top side; C—cross section; B—bottom side.

Subsequently, Col or Col-LF membranes were prepared by SBA-EPD (Figure 7a). For this purpose, previously obtained suspensions (collagen–lactoferrin or pure collagen) were used. SBA-EPD was carried out in an electrochemical cell separated by a semipermeable barrier made of regenerated cellulose (Sigma-Aldrich, St. Louis, MO, USA). Col-LF or Col suspension was poured into the anode part of the cell, and distilled water was poured into the cathode part of the cell. The cathode and anode were plate electrodes to which the DC source of 60 V was connected, and the electrodeposition process was carried out. The produced membranes were carefully separated from the surface of the semipermeable barrier and treated for 20 min with isopropyl alcohol and then dried in a laminar flow cabinet.

The post-treatment of collagen and collagen-LF membranes included their chemical cross-linking using 0.03% glutaraldehyde solution for 10 min, mechanical perforation on one side using a cosmetic mesoroller, and lyophilization for 48 h at −40 °C (Figure 7b). The post-treatment resulted in a porous (in the inner volume, cross-section) interface-type membrane with one side perforated (bottom) and the other side solid and non-perforated (top).

### 4.2. Physicochemical Membrane Characterization

Visualization of the membrane microstructure was carried out by Hitachi TM4000 scanning electron microscope (SEM) (Hitachi, Düsseldorf, Germany) at 10 kV using a back-scattered (reflected) electron (BSE) detector. To prepare the samples, they were washed with physiological saline and distilled water, cut across with a microtome blade, dried in air, and placed in the SEM chamber.

For LF release kinetics determination from collagen–lactoferrin membranes, the ELISA kit (Cloud-Clone Corp., Houston, TX, USA) was applied. For this purpose, membranes were cut into samples of equal weight. The obtained pieces were placed in PBS solution on a rotary shaker (ELMI, Riga, Latvia) at 3 RPM. The experiment was performed for 75 h, with timepoints of 2,4, 8, 20, 32, 48, 60, and 75 h (*n* = 5 samples per point).

For measuring the swelling ratio, dried samples of produced membranes (*n* = 5 for each group) were first weighed, and then placed in PBS at 4 °C for 8 h. Next, the excessive moisture from the samples was removed by filter paper, and the samples were weighed again. The swelling ratio was calculated as a change (in%) in the mass of the membrane samples according to the formula:

$$S=\frac{m_{w}-m_{d}}{m_{d}}\times 100\%$$

where *S* is the swelling ratio, *m_w_* is the mass of the wet sample, and *m_d_* is the mass of the dry sample.

The shrinkage temperature measurements were performed by the hydrothermal method with a lab-made device. For this, 20 mm × 3 mm membrane fragments (*n* = 5 for each group) were placed in a glass tube that was subsequently immersed in a water bath with distilled water. The bath temperature increased by about 5 °C/min. The temperature at which shrinkage of the membrane fragment was visually observed was determined by the thermometer.

The mechanical properties of membranes were tested in a wet environment using a Mach-1 v500csst micromechanical testing system (Biomomentum Inc., Laval, QC, Canada). The testing was performed after specimen overnight incubation with PBS at 4 °C. The modulus of elasticity (Young’s modulus, calculated in the linear region of a stress–strain curve) and strain at failure were measured in the uniaxial tension mode for at least five 30 mm × 5 mm rectangular specimens in each group (*n* = 5). Uniaxial tension was performed at a rate of 0.1 mm/s until the failure was achieved. The parameters were calculated from the deformation curves according to the manufacturer’s protocol. For measuring the dry and wet thickness of the collagen membranes, a digital caliper was applied (Mitutoyo, Tokyo, Japan).

### 4.3. Biocompatibility Tests In Vitro

To evaluate the biocompatibility of Col and Col-LF membranes in vitro, they were tested with two cell lines: keratinocytes (HaCaT line) and fibroblasts (977hTERT embryonic fibroblasts [52]). Cells were cultured using growth medium of the following composition: DMEM/F12 (1:1, BioloT, Saint Petersburg, Russia), gentamicin (50 μg/mL, PanEco, Moscow, Russia), and fetal calf serum FBS (10%, ThermoFisher, Waltham, MA, USA). Cells were seeded on membranes at a concentration of approximately 2 × 10^4^/matrix.

Cells on membranes (*n* = 5 for each group) were cultured in growth medium for 5 days. The cell metabolic activity was measured by the AlamarBlue (resazurin) assay (Invitrogen, Waltham, MA, USA) according to the manufacturer’s instructions, using a Victor Nivo spectrofluorometer (PerkinElmer, Waltham, MA, USA) at an excitation wavelength of 530 nm and emission wavelength of 590 nm. The DNA was quantified using the Quant-iT PicoGreen kit (Invitrogen, Waltham, MA, USA) according to the manufacturer’s instructions and by using a Victor Nivo spectrofluorometer at an excitation wavelength of 480 nm and emission wavelength of 520 nm. The metabolic activity of cells was also normalized to the amount of DNA.

### 4.4. Animal Experiments In Vivo

The study was conducted in accordance with the requirements of the Declaration of Helsinki and ethics rules for experimental research on animals. The protocol was approved by the Local Ethical Committee of Sechenov University (No. 06-23, 6 April 2023). Male rabbits of the “Soviet Chinchilla” breed were used for the experiments.

**Figure 8:**
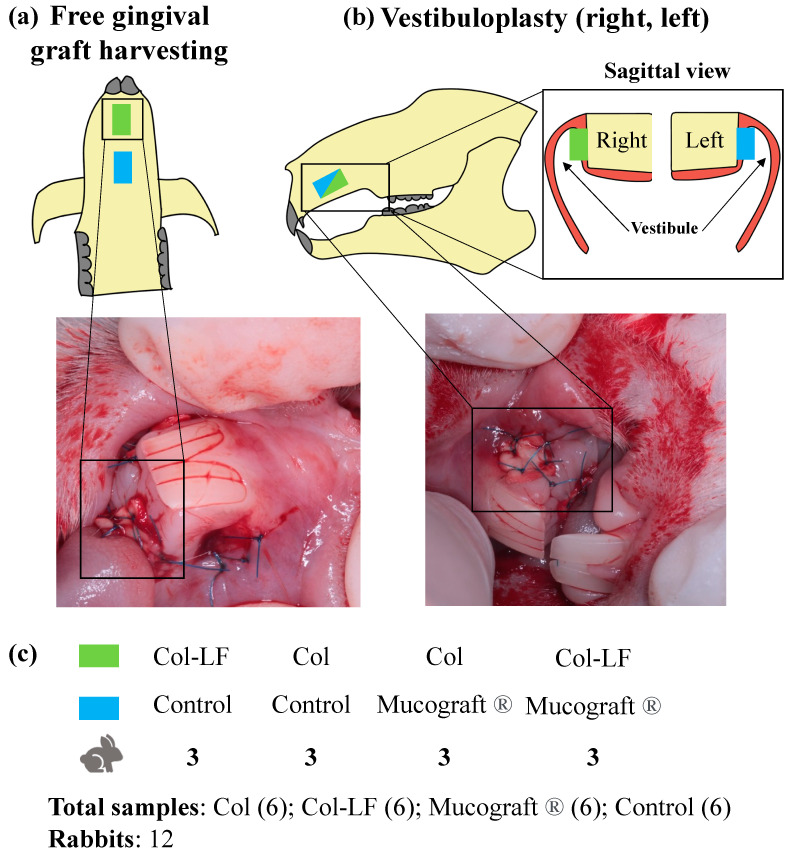
Scheme of experiments in vivo. (**a**) Free gingival graft harvesting (from hard palate); (**b**) vestibuloplasty on both right and left sides, with sagittal view of operation sites given; (**c**) four pairs of compared groups (*n* = 3 rabbits for each pair), with total *n* = 6 samples investigated in each group. Col—collagen membrane group; Col-LF—collagen–lactoferrin membrane group; Mucograft^®®^—group with commercial membrane; control—non-treated defect group.

Experimental models of free gingival graft (FGG) harvesting (Figure 8a) and vestibuloplasty (Figure 8b) were developed. The models were similar to those that can be obtained in clinical practice [53,54], since the sizes of the created wound defects in rabbits were similar to those in humans.

The rabbits were anesthetized with 1.5 mg/kg Zoletil^®^ and 0.2 mg/kg Xyla^®^ intramuscularly. During the FGG harvesting, symmetrical defects with dimensions of 0.5 cm × 0.5 cm were formed in the hard palate region (one after the other) with a scalpel. In the case of vestibuloplasty on the upper jaw (premaxilla bone), symmetrical defects with dimensions of 0.5 cm × 0.5 cm were also formed in the area of the frontal group of teeth on the left and right vestibule sides.

Subsequently, these defects were treated differently according to four study groups (Figure 8c): a group of the collagen membrane (Col); a group of the collagen–lactoferrin membrane (Col-LF); a group of the commercial membrane (Mucograft^®^, Geistlich Pharma AG, Wolhusen, Switzerland); and a group without auxiliary membranes—spontaneous healing by secondary tension (control). The fragments of 0.5 cm × 0.5 cm were cut from the investigated membranes, and they were fixed to the formed defects using Prolene 6/0 knotted sutures. Since, in animals, the entire surface of the hard palate (two surgical sites) and both sides of the maxilla were involved, it allowed combining different groups into 4 pairs in one animal: (1) Col-LF and control; (2) Col and control; (3) Col and Mucograft; (4) Col-LF and Mucograft^®^. Calculation of the required laboratory animal number for each group was performed according to formula [55]:

$$N_{target}=\frac{20}{k+1}$$

where *N* is the target number of animals in each group, *k* is the number of experimental groups.

According to our calculations, each group should have at least 4 animals (a total of 16 animals, 1 animal = 1 group). However, since two groups were paired and compared simultaneously in one animal, using 3 animals per each pair (a total of 12 animals) gave us *n* = 6 investigated samples for each group. This experimental design optimization is of great value from an experimental point of view: less animals were used in this study (ethical aspect), while more samples can be investigated in each group (6 vs. 4, statistical aspect).

**Figure 9:**
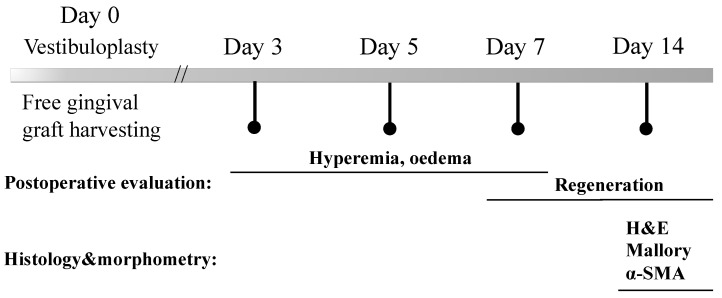
The timeline of investigations in the current study.

After FGG harvesting and vestibuloplasty, the animals were examined for 14 days (Figure 9). Intermediate postoperative examinations of the animals were performed on 3, 5, 7, and 14 days after surgery with visual assessment of oedema and hyperemia severity and defect area regeneration.

The oedema and hyperemia degrees of severity were evaluated semi-quantitatively in points relative to the initial (healthy) state (Table A1; Appendix A). Regeneration of defects was assessed quantitatively as a percentage of the total defect area by measuring with a graduated periodontal probe, ruler, and caliper. Since the created defects were initially of the same size (0.5 cm × 0.5 cm), their comparative analysis in percentages was eligible.

Animals were sacrificed on the 14th day after the operations by intramuscularly overdosing anesthesias Zoletil^®^ (15 mg/kg) and Xyla^®^ (0.6 mL/kg). After that, group samples were collected from the hard palate and oral vestibule and subjected to histological and morphometric analyses.

### 4.5. Histologic and Morphometric Analysis

The collected tissue samples (*n* = 6 in each study group) were fixed in 10% neutral buffered formalin and embedded in paraffin blocks in a strict orientation, ensuring that sections were made in a plane perpendicular to the surface of the palate or gingiva. Sections that were 3–4 µm thick were stained with hematoxylin and eosin (H&E) and Mallory trichrome. The specimens were studied by standard optical microscopy using a LEICA DM4000 B universal microscope equipped with a LEICA DFC7000 T video camera and LAS V4.8 software (Leica Microsystems, Wetzlar, Germany). Signs of inflammation (exudation, infiltration by immune cells, microcirculatory disorders) and regeneration (proliferation of fibroblasts, maturity of granulation tissue) were semi-quantitatively evaluated in each preparation according to a 4-point scale (Table A2, Table A3, Table A4, Table A5, Table A6 and Table A7; Appendix A).

For immunohistochemical analysis, 3–4 µm-thick sections of tissue samples fixed in 10% neutral buffered formalin and embedded in paraffin blocks were deparaffinized and incubated with 3% hydrogen peroxide for 10 min. Nonspecific staining was prevented by blocking solution (Cell Marque, Rocklin, CA, USA), and the samples were incubated with mouse monoclonal primary antibodies against α-smooth muscle actin, or α-SMA (A2547, Merck, Rahway, NJ, USA, dilution 1:400). Visualization was performed using horseradish peroxidase-conjugated secondary goat antibodies (G-21040, Invitrogen, USA, dilution 1: 1000) and diaminobenzidine with contrast staining with hematoxylin.

The expression of α-SMA in the implantation sites was evaluated using a semi-quantitative system (Table A7; Appendix A). For determining neoangiogenesis levels, in each sample stained with a-SMA antibodies, blood vessels were counted in 5 or more representative fields of view at ×200 magnification and calculated as a mean value. Subsequently, blood vessel density was calculated by dividing the counted number per investigated area, and the corresponding values were expressed as blood vessels/mm^2^.

### 4.6. Statistical Analysis

Statistical analysis was performed using GraphPad Prism 9.0 software (GraphPad Software Inc., La Jolla, CA, USA). The Shapiro–Wilk test was used to test the normality of data distribution. In case of normal distribution, pairwise comparison by Student’s test or two-way analysis of variance (two-way ANOVA) followed by multiple comparisons using Tukey’s test (in vitro matrix contact biocompatibility, wound regeneration at postoperative examination) were performed. In these cases, the results of statistical analysis were presented as mean ± standard deviation.

In case of discrete data, comparison was performed using the nonparametric Kruskal–Wallis test followed by multiple comparisons using Dunn’s test (postoperative assessment of hyperemia and edema, histologic, and morphometric analysis). Results were presented as median values with a range [min; max]. Differences were considered significant at *p* < 0.05.

## 5. Conclusions

In this work, we have demonstrated that the SBA-EPD method is suitable for the production of dental membranes that can be used for oral soft tissue regeneration. Particularly, we have demonstrated that a major structural protein (collagen) can be co-precipitated with other bioactive substances, such as lactoferrin, by this method. Lactoferrin, being a component of Col-LF membranes, had a positive proliferative effect on cells of epithelial and connective tissue in vitro, which proves that the selected collagen-LF ratio and LF concentrations were optimal.

Similarly, the incorporation of LF into collagen membrane had a significant positive anti-inflammatory and pro-regenerative effect on two in vivo models (FGG harvesting and vestibuloplasty), both at postoperative macroscopic examination of the oral cavity and at the level of histologic and morphometric analysis. In particular, the produced Col-LF membranes surpassed the commercial analog (Mucograft**^®^**) in a number of parameters, which allows us to declare them as a promising candidate for further clinical use for oral soft tissue regeneration.

## Figures and Tables

**Table 1:** Physical characteristics of collagen and collagen–lactoferrin membranes.

Membrane	Dry Thickness, µm	Swelling, %	Shrinkage Temperature, °C	Young’s Modulus, MPa	Strain at Fracture, %
Col	280 ± 23 *	545 ± 44	55 ± 0.5	3.2 ± 0.6	61 ± 6
Col-LF	320 ± 15	572 ± 53	54.5 ± 0.8	3 ± 0.7	59 ± 8

Abbreviations: Col—collagen membrane; Col-LF—collagen and lactoferrin membrane. Data are presented as mean ± SD. * *p* < 0.05.

## Data Availability

Data will be made available on reasonable request.

## Appendix A

**Table A1:** A scoring system for visual signs of exudation/hyperemia on postoperative examination.

Score	Signs
0	No oedema or hyperemia
1	Mild oedema/ hyperemia of the oral mucosa
2	Moderate oedema/ hyperemia of the oral mucosa
3	Prominent oedema/ hyperemia of the oral mucosa

**Table A2:** Scoring system for morphologic signs of exudation at the implantation site.

Score	Signs
0	No oedema
1	Mild signs of oedema, small amount of fluid in the intercellular space
2	Moderate signs of tissue oedema, average amount of fluid in the intercellular space
3	Prominent tissue oedema, significant amount of fluid in the intercellular space

**Table A3:** Scoring system for morphologic signs of inflammatory infiltration at the implantation site.

Score	Signs
0	No inflammation
1	Presence of single inflammatory cells in the infiltrate (less than 10 cells in 1 field of view at ×400 magnification)
2	Moderate number of inflammatory cells in the infiltrate (from 11 to 29 cells in 1 field of view at ×400 magnification)
3	Large number of inflammatory cells in the infiltrate (more than 30 cells in 1 field of view at ×400 magnification)

**Table A4:** Scoring system for morphologic signs of microcirculatory disorders in the implantation site.

Score	Signs
0	No signs of microcirculation disorders
1	Marginal standing (wall-to-wall) of erythrocytes in the vascular lumen
2	Initial manifestations of erythrocyte aggregation and agglutination in the vascular lumen
3	Stasis and sluggishness of erythrocytes in the vascular lumen

**Table A5:** Scoring system for morphologic signs of fibroblast proliferation at the implantation site.

Score	Signs
0	No signs of fibroblast proliferation
1	Mild signs of hypertrophy and hyperplasia of fibroblasts, increase in their volume by less than 10%
2	Moderate signs of hypertrophy and hyperplasia of fibroblasts, increase in their volume by 20–30%
3	Prominent signs of hypertrophy and hyperplasia of fibroblasts, increase in their volume by more than 30%

**Table A6:** Scoring system for morphologic signs of granulation tissue maturity at the implantation site.

Score	Signs
0	No granulation tissue
1	There is young granulation tissue with abundant signs of vascularization
2	There are thin bundles of connective tissue fibers and small numbers of vessels in the granulation tissue
3	Granulation tissue is practically absent, but there is a mature connective tissue formed

**Table A7:** Scoring system for evaluating the expression of antibodies against α-SMA.

Score	Signs
0	No expression
1	Individual positively stained cells
2	Small number of positively stained cells (less than 19 per 1 field of view at a magnification of 400)
3	Significant number of positively stained cells (more than 20 per 1 field of view at a magnification of 400)
